# Turning over New Leaves

**Published:** 1888-01-28

**Authors:** 


### TURNING OVER NEW LEAVES.

[Books for Review should be sent to The Editor, The Lodge, Porchester Square, W.]

### TO OUR READERS.

FOR the future, notices of books and other reviews will appear in the last weekly issue of each month. THE HOSPITAL has now become a popular family and institutional journal, and is very widely read by many classes of people. All books, magazines, and pamphlets should reach the Editor of THE HOSPITAL by the middle of each month at above address.

### Descriptive Anatomy.*

THE eleventh edition of this well-known work has been considerably improved.

In the section on Osteology, the dotted lines on the bones indicating the attachment of muscles have been coloured, and care has been taken not to confuse the beginner by any arbitrary division as to which is the origin or insertion of a muscle. The time of appearance of the various centres of ossification has been revised; chief among the changes is that the sphenoid is said to arise from fourteen instead of ten centres. Formerly, the anterior part of the body and the lesser wing were described as springing from a common point; but in this edition a separate centre is assigned to each, and one also for the lingula, for the first time in this work.

The paragraph on the movements permitted in the knee-joint has been re-written, and due weight is given to Professor Humphry's views on the subject. In describing the mechanism of the wrist-joint, the editor has wisely omitted Meyer's complicated account of dorsi-flexion. "Development" has been re-written, and is well up to date. When we consider, however, the difficulty of the subject, and the changes this branch of science is constantly undergoing, we are not sure that it would not be better to omit the section entirely in future editions, and to refer the advanced student to systematic works on the subject. The space so gained might be well employed in enlarging the paragraphs on "Surgical Anatomy," which omit much matter of great value to the future surgeon. For example, there is no mention of the changes which can be induced in the position of the bladder by distension of the rectum. Now that the bladder is so frequently explored from above, it would be well to call attention to Garson's well-known investigations. We are also disappointed at finding no description of the relations between the cerebral convolutions and the cranium, a branch of anatomy rapidly becoming of great importance.

* "Gray's Anatomy; Descriptive and Surgical." Eleventh edition. Edited by J. Pickering Pick, F.R.C.S., Surgeon to St. George's Hospital, Member of the Court of Examiners, Royal College of Surgeons.

In the description of the minute anatomy of the small intestine we notice that the editor accepts Watney's theory as to the absorption of fat, in preference to those of Schäfer or the older histologists.

The illustrations, which have always been a strong point in this work, have been rendered clearer by judicious colouring; the arteries, veins, and nerves being tinted red, blue, and yellow respectively; the diagram by Dr. Delépine, showing the lines along which the peritoneum leaves the wall of the abdomen to invest the viscera, being particularly good.

In the description of the cranial nerves, the auditory nerve—the three origins of which are given—is said to subsequently divide into cochlear and vestibular branches, the latter being distributed to the vestibule and semi-circular canals; we do not, however, find it mentioned that the fibres of origin from the *cerebellum* pass to the semi-circular canals alone, and that none of these fibres go to form the cochlear branch. Dr. Gowers has shown the importance of this anatomical point in the diagnosis of cere bellar disease.

We have called attention to one or two of the omissions which still mar the excellence of this work, and we must again urge the editor in his next issue to rewrite the sections relating to surgical anatomy and operative surgery. But, as a whole, this edition is greatly superior to the last, and will enable the work to retain its high position as a text-book on anatomy, whilst it will add to the reputation of its editor as a sound and practical anatomist.

"Gray's Anatomy" is still, we think, the most trustworthy book on the subject that can be placed in the hands of a student.

### Work for the Blind.*

THIS most interesting volume tells what a blind woman did for her fellow-sufferers. Elizabeth Gilbert, daughter of Dr. Gilbert, first Principal of Brasenose College, Oxford, and afterwards Bishop of Chichester, lost her sight at the age of three years after an attack of scarlet fever. In spite of her affliction her father determined that her education should proceed on exactly the same lines as that of her seeing sisters. No carelessness of behaviour, no inferiority of manners was permitted because of her blindness; even when the child fell in front of the fire and tried to save herself by grasping the hot bars of the grate, Dr. Gilbert did not flinch from his determination to make her in all respects as independent as possible. Nevertheless, he must have been moved by the pleas the little girl uttered before she grasped the nature of her loss—"Take me out of the dark room"; and "If I am a *very* good 'ittle girl, may I see my dolly to-morrow?"

The result of this was that Miss Gilbert grew up a clever and cultured woman, superior to other young ladies rather than the reverse. She had, too, a remarkably energetic temperament, which led her to inquire into the general method of instruction to the blind, and to do her best to improve it. She found that the institutions for the blind provided instruction for the young, and for them only. And again she learnt that the existing institutions dismissed young men and women who had been fairly educated and taught a trade, on the assumption that as adults they could practise their trade and earn a living. This conjecture tells cruelly on the blind. The blind men and women cannot go about from place to place in search of work, and have no market for their goods if they work at home. "To overcome this, Miss Gilbert in 1854 hired a cellar in New Turnstile, Holborn, at the cost of 1*s*. 6*d*. a week, for the sale of the work of seven blind men who worked at their own homes. These were paid the full price of their work, less only the cost of material. The attempt prospered, and became the first beginning of the institution known as 'The Association for Promoting the General Welfare of the Blind.'"

* "Elizabeth Gilbert and her Work for the Blind." By Frances Martin. London: Macmillan and Co.

Miss Gilbert's coadjutor in this work was Mr. Hanks Levy, who was also blind, and was originally a teacher in the St. John's Wood School. He travelled on behalf of the institution, made investigations, and transacted business with a vigour, intelligence, and success which would have been remarkable in a man possessed of all his faculties. Without going farther into the contents of this book, we may say that it is full of value as showing to how small a degree the blind need be separated from others in the active work of life, and as showing that they need not because of their misfortune give up one iota of independence or usefulness.

### Health for the People.*

EXCEPT as indicating that the articles reprinted in it have originally appeared in *Health*, the title of Dr. Andrew Wilson's volume must be regarded as misleading. The book is not devoted, as its name would imply, to the study of those sanitary laws which it is everybody's interest—if not everybody's duty—to have some knowledge of. These are, indeed, brought forward here and there, but for the most part the volume deals with the curiosities of physiology, pathology, and medicine, touching in a pleasant, desultory manner on old and new fashions and caprices in treatment, and discussing medical superstitions of ancient and modern times. On these subjects Dr. Wilson discourses pleasantly, and no doubt his talks about memory, disease and art, meat teas, and hair-dyes will interest many readers.

### The Ministry of Nature.†

MR. GILBART-SMITH is a disciple of two poets of such different tone as Rossetti and Lewis Morris. Akin to the first is the seductive melody of his ryhthm and his skilful use of alliteration, while his ethical code is allied to that of the second. The central idea of "Serbelloni" is the ministry of Nature to the soul of man, that recognition of the existence of God which comes from appreciation of natural beauty. This is elaborately developed, and gives scope for many passages of beautiful description, in which the author excels. Doubtless, however, he himself values most the metaphysical argument that runs throughout the volume; though we venture to think this of less artistic value. The creed enunciated therein is indeed that of a thoughtful, sincere, and earnest man; but it is not one that tends greatly to clear away the doubts that obscure the vision of our day; and in many passages the very smoothness and beauty of the style militates against vigour of utterance. Carlyle and Browning have taught us to value those thoughts that come red-hot from the writer's soul—things felt as well as perceived; in Mr. Gilbart-Smith's verses the fusing-point of heart and mind is either passed or not attained.

### The English Illustrated Magazine.

MESSRS. MACMILLAN have done wonders with this magazine, and it may fairly be described as the cheapest sixpenny monthly. The letterpress is seldom or never dull, and most of it is excellent, the interest of many of the stories being well sustained and attractive. The illustrations are spirited, and display a mastery of detail and an excellence of execution rarely met with in periodicals of this description. The "English Illustrated Magazine" is a general favourite, and its circulation will no doubt increase as its many excellencies are known and appreciated.

* "Health for the People." By Andrew Wilson, F.R.G.S. London: Sampson Low, Marston, Searle, and Rivington.

† "Serbelloni: a Poem." By J. W. Gilbart-Smith. London: Kegan Paul, Trench, and Co.

